# Structural and functional connectivity from the dorsomedial hypothalamus to the ventral medulla as a chronological amplifier of sympathetic outflow

**DOI:** 10.1038/s41598-020-70234-4

**Published:** 2020-08-07

**Authors:** Yosuke Kono, Shigefumi Yokota, Isato Fukushi, Yosuke Arima, Hiroshi Onimaru, Shuntaro Okazaki, Kotaro Takeda, Itaru Yazawa, Masashi Yoshizawa, Yohei Hasebe, Keiichi Koizumi, Mieczyslaw Pokorski, Takako Toda, Kanji Sugita, Yasumasa Okada

**Affiliations:** 1 Department of Pediatrics, Faculty of Medicine, University of Yamanashi, Chuo, Yamanashi 409-3898 Japan; 2 Clinical Research Center, Murayama Medical Center, 2-37-1 Gakuen, Musashimurayama, Tokyo 208-0011 Japan; 3 Department of Anatomy and Morphological Neuroscience, Shimane University School of Medicine, Izumo, Shimane 693-8501 Japan; 4 Faculty of Health Sciences, Uekusa Gakuen University, Chiba, 264-0007 Japan; 5 Department of Physiology, Showa University School of Medicine, Shinagawa, Tokyo 142-8555 Japan; 6 Faculty of Human Sciences, Waseda University, Tokorozawa, Saitama, 359-1192 Japan; 7 Faculty of Rehabilitation, School of Healthcare, Fujita Health University, Toyoake, Aichi 470-1192 Japan; 8 Global Research Center for Innovative Life Science, Hoshi University, Shinagawa, Tokyo 142-8501 Japan; 9 Faculty of Health Sciences, University of Opole, 45-060 Opole, Poland

**Keywords:** Neuroscience, Physiology

## Abstract

Psychological stress activates the hypothalamus, augments the sympathetic nervous output, and elevates blood pressure via excitation of the ventral medullary cardiovascular regions. However, anatomical and functional connectivity from the hypothalamus to the ventral medullary cardiovascular regions has not been fully elucidated. We investigated this issue by tract-tracing and functional imaging in rats. Retrograde tracing revealed the rostral ventrolateral medulla was innervated by neurons in the ipsilateral dorsomedial hypothalamus (DMH). Anterograde tracing showed DMH neurons projected to the ventral medullary cardiovascular regions with axon terminals in contiguity with tyrosine hydroxylase-immunoreactive neurons. By voltage-sensitive dye imaging, dynamics of ventral medullary activation evoked by electrical stimulation of the DMH were analyzed in the diencephalon-lower brainstem-spinal cord preparation of rats. Although the activation of the ventral medulla induced by single pulse stimulation of the DMH was brief, tetanic stimulation caused activation of the DMH sustained into the post-stimulus phase, resulting in delayed recovery. We suggest that prolonged excitation of the DMH, which is triggered by tetanic electrical stimulation and could also be triggered by psychological stress in a real life, induces further prolonged excitation of the medullary cardiovascular networks, and could contribute to the pathological elevation of blood pressure. The connectivity from the DMH to the medullary cardiovascular networks serves as a chronological amplifier of stress-induced sympathetic excitation. This notion will be the anatomical and pathophysiological basis to understand the mechanisms of stress-induced sustained augmentation of sympathetic activity.

## Introduction

Psychological stress activates the sympathetic nervous system and elevates arterial blood pressure^1^. When psychological stress arises, it is sensed and processed by the cerebral cortex and limbic system, and the information is relayed to the hypothalamus via the central nucleus of the amygdala^2–4^. The hypothalamus, especially the dorsomedial hypothalamus (DMH), plays a crucial role in mediating and processing the cardiovascular responses to acute psychological stress^5^. It has been known that hypothalamic excitation activates the rostral ventrolateral medulla (RVLM), medullary raphe regions, and intermediate lateral cell column of the spinal cord, augmenting sympathetic activity, heart rate, and blood pressure^6–8^. The hypothalamus is essential for the occurrence of hypertension via the hypothalamic–pituitary–adrenal and sympathetic-adrenomedullary axes^9^, and indeed chronic electrical stimulation of the hypothalamus induces hypertension in animal experiments^10^. However, anatomical and functional connectivity from the hypothalamus to the medullary cardiovascular regions has not been fully clarified. Therefore, this study seeks to define the anatomical and functional connectivity from the DMH to the medulla to clarify the neural substrate mediating the propagation of stress-induced sympathetic activity and to gain insight into the pathophysiology of hypertension.

## Materials and methods

### Retrograde tract-tracing

The experimental procedures were akin to those described previously^11^. Young adult male Wistar rats (8–10-week old, n = 3) were anesthetized with intraperitoneal chloral hydrate (350 mg/kg). Fluorogold (FG) (Fluorochrome, Denver, CO) (5% dissolved in saline) was iontophoretically injected into the RVLM. We inserted a pipette to the medulla caudally tilting at a 60-degree angle from the dorsal surface at the point 0.5 mm caudal to the obex and 2.1 mm right of the midline. We advanced the pipette by 2.8 mm in this direction. A driving current (3 μA, 400 ms, 1 Hz) was delivered for 15–20 min. After 7–10-day survival, rats were deeply anesthetized with a lethal dose of chloral hydrate (700 mg/kg) and transcardially perfused with saline followed with 4% paraformaldehyde in 0.1 mol/L phosphate buffer (PB, pH 7.3). Then, the brains were removed, postfixed, saturated with 20% sucrose in the same buffer, and cut into frontal 30 μm thick sections on a freezing microtome. The sections were incubated in the blocking buffer (0.1 mol/L phosphate buffered saline, pH 7.3; PBS containing 0.3% Triton-X and 3% normal donkey serum) for 1 h, and further incubated overnight in the buffer containing guinea pig anti-FG antibody (1:1,000; Protos Biotech, New York, NY). Subsequently, the sections were further incubated in the buffer containing biotinylated anti-guinea pig IgG (1:500; Jackson ImmunoResearch, West Grove, PA) for 3 h, followed by PBS containing 0.3% Triton-X and ABC-Elite (1:500; Vector Labs., Burlingame, CA) for 1 h, and finally in 25 mL of 0.1 mol/L PB containing 10 mg diaminobenzidine (DAB; Nacalai Tesque, Kyoto, Japan) and 10 µL of 30% hydrogen oxide. After incubation, sections were mounted onto gelatinized slides, coverslipped, and examined under a light microscope (Eclipse E800; Nikon, Tokyo, Japan). FG-labeled neurons in the hypothalamus were plotted using a camera lucida. Coverslips were then removed, and the sections were counterstained with 1% cresyl violet for cytoarchitectural landmarks.

### Anterograde tract-tracing

Anterograde tracing was performed in male Wistar rats (8–10-week old, n = 5) anesthetized with a mix of anesthetic agents (0.3 mg/kg of medetomidine, 4.0 mg/kg of midazolam, and 5.0 mg/kg of butorphanol, i.p.). The tracer, biotinylated dextran amine (BDA) (Molecular Probes, Eugene, OR) (10% dissolved in 0.01 mol/L PB) was stereotaxically injected into the DMH as a single iontophoretic injection (5 µA, 400 ms, 1 Hz, 30 min)^11^. For the injection of BDA a pipette tip was placed at the site with its coordinates 2.6 mm caudal to the bregma, 0.4 mm right of the midline and 8.6 mm deep from the dorsal surface of the brain. After 1-week survival, the rats were deeply reanesthetized and perfused. Then, the brains were removed, postfixed, saturated with a 20% sucrose solution, and cut on a freezing microtome as described above.

One of every two series of sections was incubated in PBS containing 0.3% Triton X-100 and ABC-Elite for 1 h, followed by incubation in the solution consisting of 1.25 µmol/L biotinylated tyramide, 3 µg/mL of glucose oxidase, 2 mg/mL of beta-D-glucose, and 1% bovine serum albumin in 0.1 mol/L PB to intensify the BDA signals. Subsequently, sections were incubated in ABC-Elite for 1 h and then treated with ImmPACT SG solution (Vector Labs). BDA-labeled axons were stained dark blue to black. Finally, the sections were mounted, coverslipped, examined under a light microscope and photographed with a virtual slide scanner (PreciPoint, M8). BDA-labeled axons and terminals were plotted on photographs using a camera lucida.

### Combination of anterograde tracing with immunohistochemistry for tyrosine hydroxylase

The anatomical relationship of DMH axon fibers and tyrosine hydroxylase (TH)-immunoreactive neurons in the medulla was investigated in another series of sections from 2 rats. When the BDA-labeled axons were visualized as described above, the sections were incubated overnight in avidin blocking solution (Avidin/Biotin Blocking Kit; Vector Labs) and then incubated in mouse monoclonal antibody for TH (1:2000; Immunostar, Hudson, WI) in biotin solution (Avidin/Biotin Blocking Kit) for 24 h. Subsequently, sections were incubated in biotinylated donkey anti-mouse IgG, incubated in ABC-Elite, and then reacted with DAB solution. They were mounted, coverslipped, and photographed using the light microscope.

### In vitro preparation for voltage imaging

**Figure 1:**
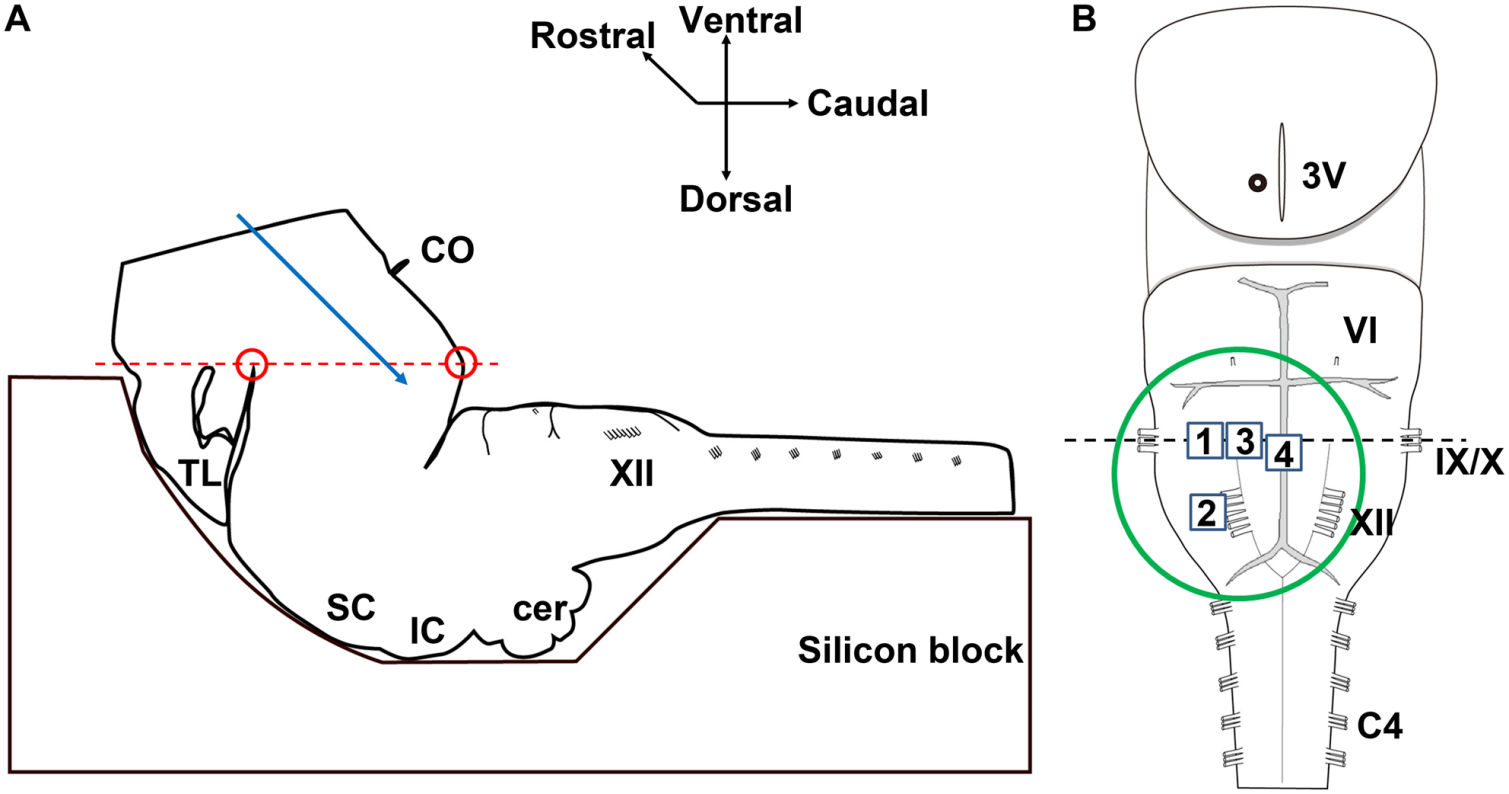
Scheme of the isolated diencephalon-lower brainstem spinal cord preparation. (**A**) Lateral view of the preparation before resection of rostral brain structures. The preparation was transected at the level between the hypothalamic infundibulum and the bilateral basal regions of the temporal lobe (both points are shown with red circles, and the cut line is shown with a red dashed line). The cut-surface, with the ventral medulla facing up, was fixed with miniature pins onto a silicon block. A blue arrow shows the direction of microelectrode insertion. (**B**) Ventral view of the preparation after resection of brain structures rostral to the cut-line indicated in panel A. This preparation included a part of the hypothalamus, midbrain, pons, medulla and cervical spinal cord. The green circle marks the area of recordings. Fluorescence change was quantified in four squared areas. Square 1: rostral ventrolateral medulla (RVLM), Square 2: caudal ventrolateral medulla (CVLM), Square 3: rostral ventromedial medulla (RVMM), Square 4: raphe pallidus (RP). The insertion point of the stimulating microelectrode on the cut surface is shown with a black circle. C4, ventral roots of the fourth cervical spinal cord; cer, cerebellum; CO, optic chiasm; IC, inferior colliculus; SC, superior colliculus; TL, part of the temporal lobe; VI, sixth cranial nerve roots; IX, ninth cranial nerve roots; X, tenth cranial nerve roots; XII, twelfth cranial nerve roots; 3 V, third ventricle.

We used a diencephalon-lower brainstem-spinal cord preparation obtained from neonatal Wistar rats (either male or female, 0–2-day old, n = 9). The principal procedure for preparing the preparation has been previously described^12,13^. In brief, under deep isoflurane anesthesia, the brain and spinal cord were quickly isolated. The temporal lobes and other cerebral structures were removed. Then, the structures rostral to the level between the hypothalamic infundibulum and the bilateral basal regions of the temporal lobe were dissected (Fig. 1A). The dissection took place in the artificial cerebrospinal fluid (aCSF) consisting of (mmol/L) NaCl 124, KCl 5, KH_2_PO_4_ 1.2, CaCl_2_ 2.4, MgCl_2_ 1.3, NaHCO_3_ 26_,_ and glucose 30, equilibrated with 95% O_2_ and 5% CO_2_ (pH 7.4). For the staining with a voltage-sensitive dye, the preparation was incubated in aCSF containing a voltage-sensitive dye di-2-ANEPEQ (0.15–0.2 mmol/L) (Invitrogen, Carlsbad, CA) for 50 min while bubbling 95% O_2_ and 5% CO_2_ gas mixture^14–16^. Dissection of the preparation and its staining with the voltage-sensitive dye was conducted in aCSF at room temperature of 24–25 °C.

### Electrical stimulation and optical recording

After staining, the diencephalon-brainstem-spinal cord preparation was transferred into a recording chamber, mounted under an epifluorescence microscope (Optiphoto-2-UD, Nikon, Tokyo, Japan) with a 4 × objective lens (Plan Apo, NA 0.20, Nikon). Then, the preparation was fixed on the chamber floor, the ventral side up, with miniature pins, and was superfused with oxygenated aCSF of 26–28 °C at a rate of 4 mL/min^13,17^. The preparation’s viability was confirmed by monitoring neural respiratory output from the C4 ventral root of the cervical spinal cord (Supplementary Fig. 1).

To stimulate the hypothalamus, a monopolar tungsten microelectrode (catalog# 573,400, A-M Systems, Carlsborg, WA) was stereotaxically inserted from the transected surface of the diencephalon, so that its tip was within the DMH region, according to a neonatal rat brain atlas^18^. The insertion point was set 0.3–0.5 mm lateral to the midline (third ventricle) and 0.1 mm deep from the surface (Fig. 1B). The microelectrode position was periodically checked, and adjusted when necessary, so that the electrically stimulated region coincided with the DMH, by confirming the concordance between the directly stimulated and depolarized area monitored by voltage imaging. Electrical stimulation was performed with two different modalities. One was a single pulse of 0.5–1 mA intensity and 3–5 ms duration, which were conducted in 5 preparations. Among these 5 preparations, four were used only in single pulse stimulation experiments. The stimulation intensity and duration were slightly adjusted depending on the responsiveness in each preparation. The other was tetanic stimulation of 0.4 mA intensity, 500 μs pulse duration, 100 Hz frequency, and 10 s tetanic stimulation duration, which was conducted also in 5 preparations. Among these 5 preparations four were used only in tetanic stimulation experiments, and one for both single pulse and tetanic stimulation experiments.

To detect stimulation-induced neural activation, fluorescence signal in the ventral medulla was continuously captured by optical recording system. The imaged ventral medullary area is indicated with a circle in Fig. 1B. The preparation was illuminated with a tungsten-halogen lamp (150 W) through a band-pass excitation filter (λ = 480–550 nm), and epifluorescence was detected through a long-pass barrier filter (λ > 590 nm) with a CMOS sensor array (100 × 100 μm pixel size, 100 × 100 pixel array; MiCAM Ultima, BrainVision, Tokyo, Japan)^11^. The optical signal was sampled at a frame rate of 50 Hz in single pulse stimulation and 12.5–25 Hz in tetanic stimulation. In the former mode of stimulation, optical signal was recorded for 5.12 s, starting at 1.28 s before the onset of stimulation. In the latter, the optical signal was recorded for 40.96 s, starting at 10.24 s before the onset of stimulation.

### Statistical analysis

Imaging data were analyzed using an image analysis software, BV Analyzer (BrainVision). Details of imaging data analyses were as we described previously^11,14–16,19^. A change in fluorescence intensity (ΔF) relative to initial intensity (F0) in each pixel was calculated. To normalize the difference in the amount of membrane-bound dye and illumination within the preparation, background fluorescence intensity at each pixel was divided by the maximum background fluorescence. Then, ratio of ΔF to the normalized background fluorescence intensity (F) was calculated at each pixel in each frame (ΔF/F). If F was less than 0.25, ΔF⁄F was set to zero. A negative ΔF⁄F corresponds to membrane depolarization. To increase the signal-to-noise ratio, cycle triggered averaging was conducted for 20–50 cycles with 7–20 s intervals in the single pulse stimulation and 5 cycles with 1.5–5 min intervals in tetanic stimulation. To evaluate the depolarizing neural activity in the ventral medulla evoked by hypothalamic stimulation, changes in epifluorescence were quantified at four squared areas in Fig. 1B (each 0.5 mm × 0.5 mm), i.e., ipsilateral RVLM, ipsilateral caudal ventrolateral medulla (CVLM), ipsilateral rostral ventromedial medulla (RVMM), and the raphe pallidus (RP). We determined these regions at the level of the XIIth cranial nerve root—1.3 mm lateral to the midline, the XIIth cranial nerve root—0.3 mm lateral to its root, the same level of the RVLM—0.5–1.0 mm lateral to the midline, and in the midline area at the level between the Xth and XIIth cranial nerve roots, respectively. Stimulation-induced excitation was defined as a median value of ΔF/F twice larger than the baseline value. The duration of excitation was used as an index of post-stimulus 75% recovery time, which was defined between the endpoint of stimulation and the point of a median ΔF/F decay by 75% off its peak. Data were presented as means ± SE. Differences between post-stimulus 75% recovery time in the single pulse and tetanic stimulation were analyzed with the Welch *t*-test. A *p* value < 0.05 defined statistically significant differences.

### Ethics approval

All experiments were carried out in accordance with the National Institutes of Health Guide for the Care and Use of Laboratory Animals (NIH Publications No. 80-23) revised 1996. Experimental protocols were approved by the Ethics Committees for Animal Research of Murayama Medical Center and Shimane University. All efforts were made to minimize the number of animals used and their suffering.

## Results

### Histology

**Figure 2:**
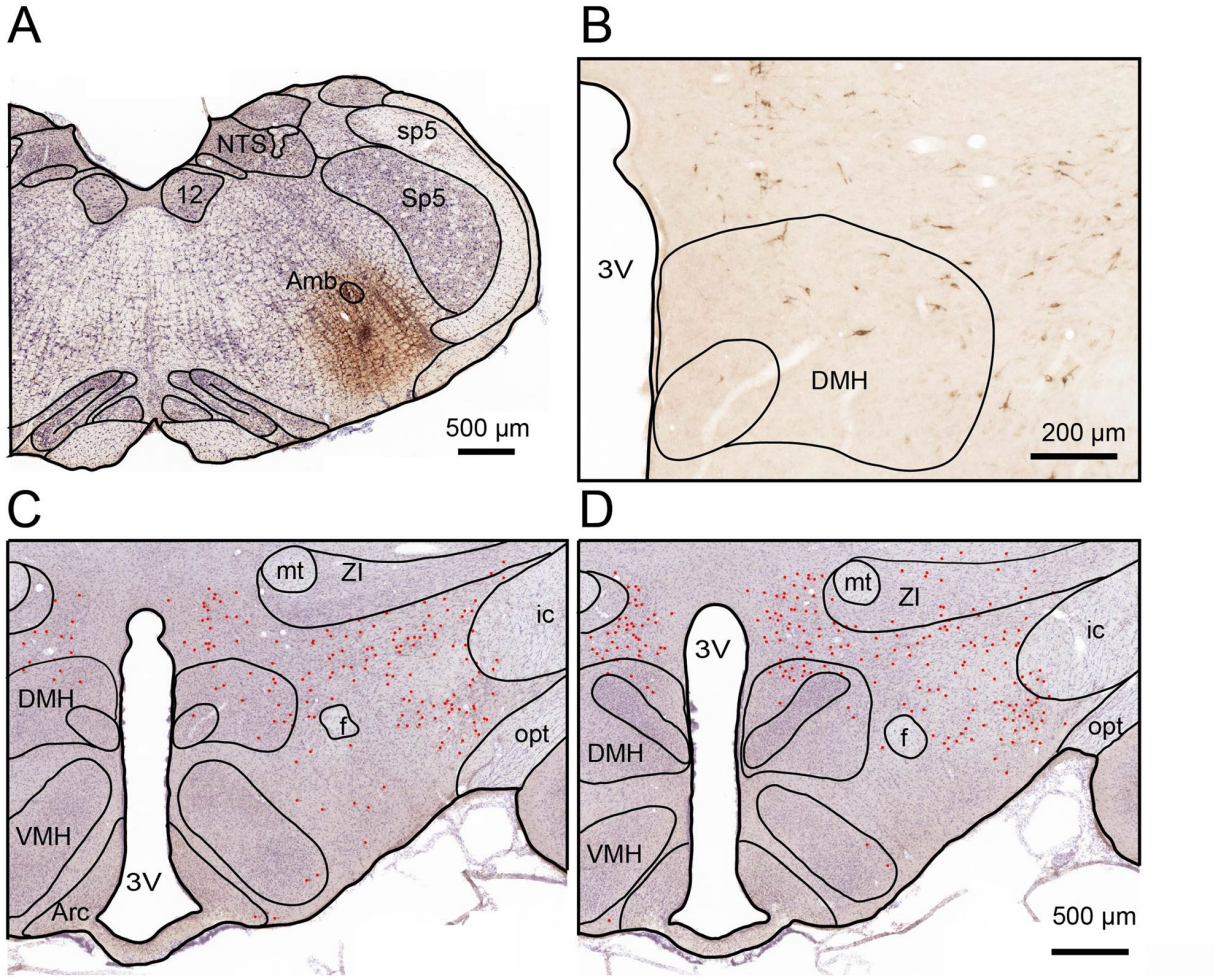
Distribution of RVLM-projecting neurons in the DMH. A color photomicrograph showing the site of FG injection into the RVLM (**A**), and retrogradely labeled neurons with FG in the DMH (**B**). (**C**, **D**) The distribution of FG-labeled neurons in two levels of the hypothalamus which contains the DMH (**C**, **D**; rostral to caudal). Each red dot indicates one FG-labeled neuron. Note that labeled cell bodies of neurons that projected to the RVLM were found in the DMH and adjacent regions in the hypothalamus. Arc, arcuate nucleus of the hypothalamus; DMH, dorsomedial hypothalamus; f, fornix; VMH, ventromedial hypothalamus; ZI, zona incerta; 3 V, third ventricle.

We first examined the distribution of RVLM-projecting neurons in the DMH, using a retrograde tracing technique by injection of FG into the RVLM. The accuracy of the injection site was confirmed by histologically observing that the FG deposit was limited in the RVLM (Fig. 2A). Retrogradely FG-labeled neurons were observed bilaterally in the hypothalamus with a clear-cut ipsilateral dominance. At the level of the DMH, these neurons were distributed sparsely in the tuberal hypothalamus dorsal and lateral to the fornix, in the peduncular part of the lateral hypothalamus, and in the posterior hypothalamic nucleus (Fig. 2, Supplementary Fig. 2).

**Figure 3:**
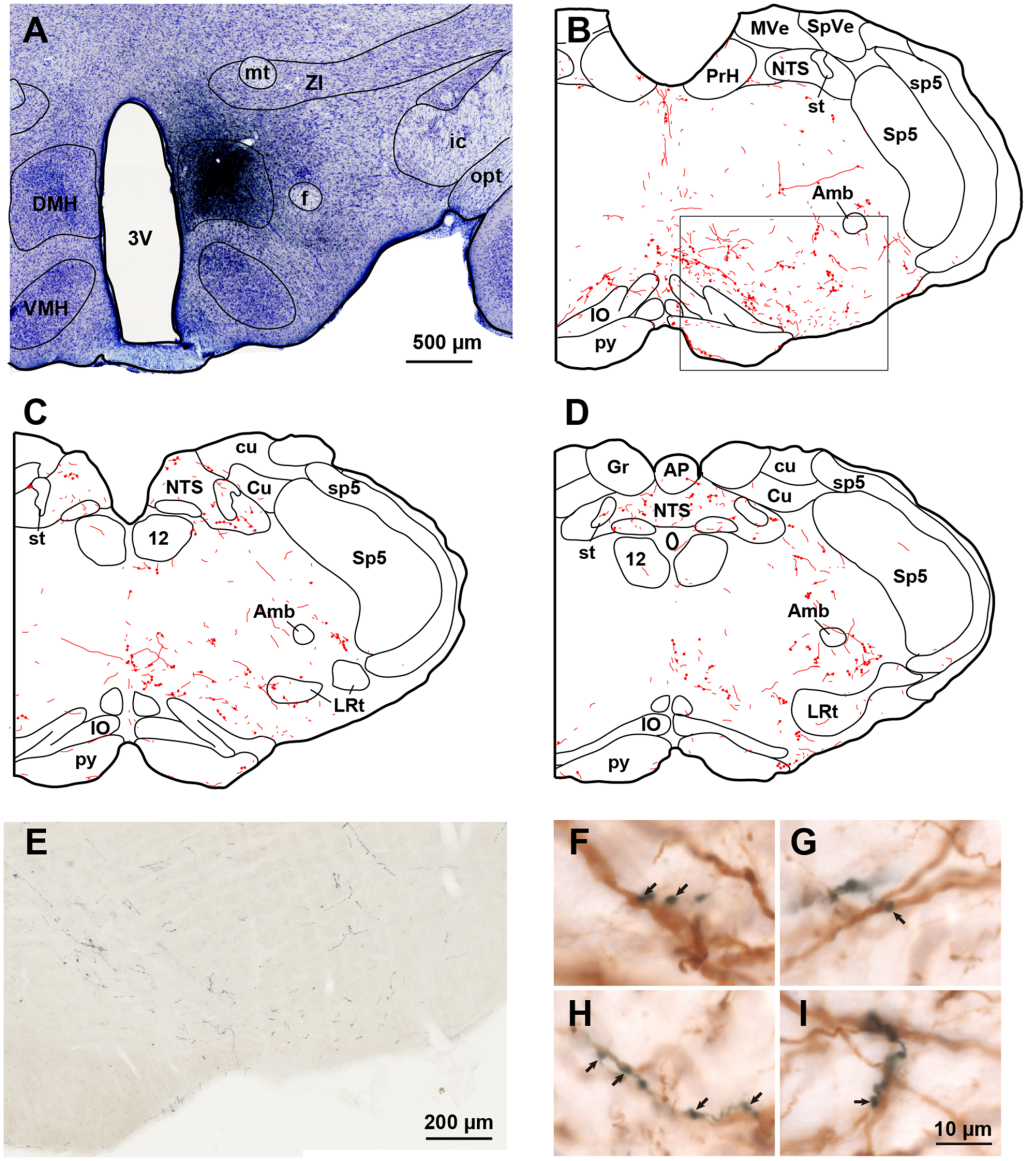
A photomicrograph showing the site of BDA injection into the DMH (**A**), and line drawings showing the resulting distribution of BDA-labeled axon fibers (red fine lines) and terminals (red fine dots) in the medulla oblongata (**B**–**D**; rostral to caudal). (**E**) Distribution of anterogradely labeled axon fibers (black-colored) in the ventromedial and ventrolateral medulla. (**F**–**I**) Overlapping distribution of BDA-labeled axon fibers and TH-immunoreactive neurons in the RVLM. Note that the BDA-labeled boutons (arrows) are in contiguity with dendrites of the TH-immunoreactive neurons. Such close apposition was frequently observed. Amb, nucleus ambiguus; AP, area postrema; Cu, cuneate nucleus; Gr, gracile nucleus; ic, internal capsule; IO, inferior olivary nucleus; LRt, lateral reticular nucleus; MVe, medial vestibular nucleus; NTS, nucleus of the solitary tract; opt, optic tract; PrH, prepositus hypoglossal nucleus; py, pyramidal tract; SpVe, spinal vestibular nucleus; Sp5, spinal trigeminal nucleus; sp5, spinal trigeminal tract; st, solitary tract; TH, tyrosine hydroxylase; 12, hypoglossal nucleus. For other abbreviations, see Fig. 2 legend.

We further examined the neuronal projection from the DMH to the medulla oblongata by anterograde tracing. In two rats that were most optimally injected with BDA into the DMH, in which the BDA deposit was confined to the DMH (Fig. 3A, Supplementary Fig. 3), anterogradely BDA-labeled fibers and axon terminals were distributed bilaterally, also with a clear-cut ipsilateral dominance, in the reticular formation of the medulla oblongata. In addition, a few labeled axons were seen in the rostrocaudal extent of the nucleus of the solitary tract (Fig. 3B–D). Many labeled fibers were found in the ventromedial medulla, which extended to the mid-medullary region. Also, a few labeled axons were present in the ventrolateral medulla, on the surface of the pyramidal tract, and at the boundary between the pyramidal tract and ventromedial medulla (parapyramidal region) (Fig. 3B–E). In the RVLM, there is a cluster of tyrosine hydroxylase (TH)-immunoreactive catecholaminergic neurons. We found bouton-like varicosities labeled with BDA were in close apposition to the dendrites of TH-immunoreactive neurons (Fig. 3F–I, Supplementary Fig. 4).

**Figure 4:**
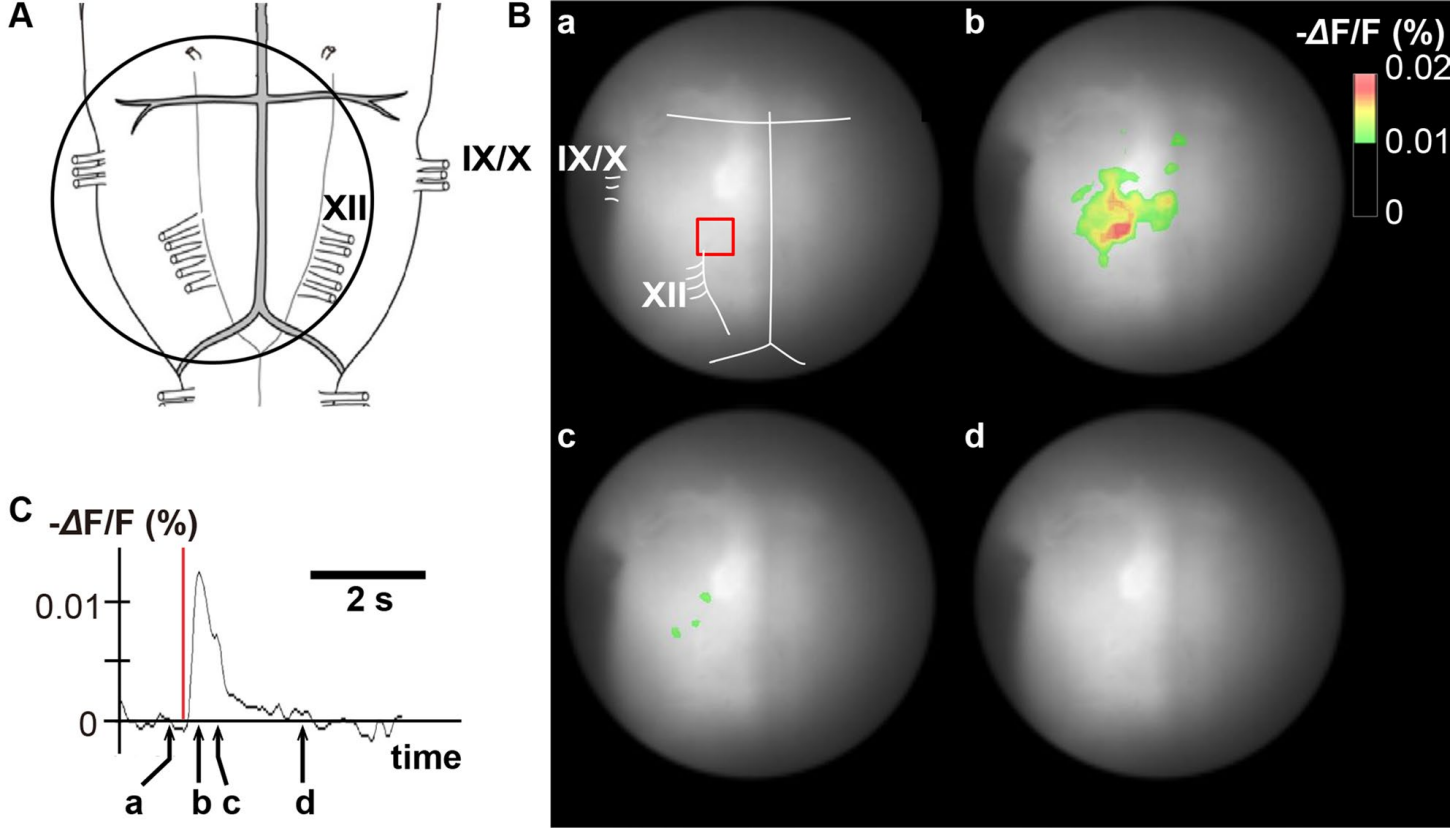
Representative optical images in a single pulse stimulation experiment. (**A**) The circle denotes the recording area on the ventral medulla. (**B**) Pseudocolor images showing fluorescence intensity on the ventral medulla before, during, and after single pulse stimulation. (**C**) Time course of fluorescence intensity in the red square area of panel B-a. A vertical red bar shows the stimulation period. **a**: before stimulation, **b**: 200 ms after the end of stimulation, **c**: 600 ms after the end of stimulation, **d**: 2.0 s after the end of stimulation. IX, ninth cranial nerve roots; X, tenth cranial nerve roots; XII, twelfth cranial nerve roots.

### Voltage imaging

Depolarizing neural activities on the ventral medulla induced by single pulse and tetanic electrical stimulation of the DMH were visualized by voltage imaging. The accuracy of the stimulating microelectrode placement was histologically confirmed by observing that the damaged region corresponded to the DMH (Supplementary Fig. 5). A representative image of the single pulse stimulation of the hypothalamus is shown in Fig. 4. This stimulation induced depolarizing optical signals on the RVLM and RVMM in all specimens, whereas activation of the CVLM was detected in three and that of the RP in four out of the five specimens, respectively.

**Figure 5:**
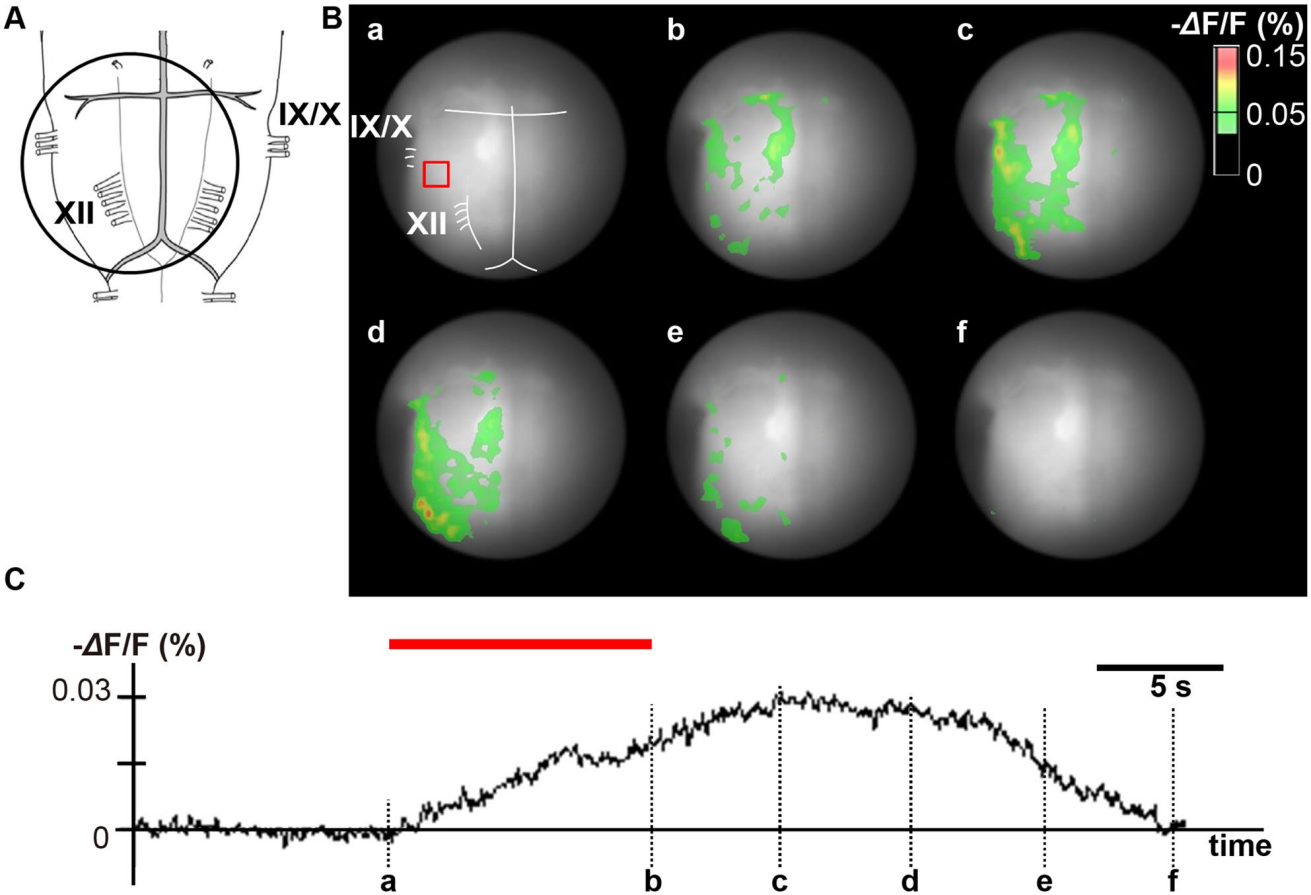
Representative optical images in a tetanic stimulation experiment. (**A**) The circle denotes the recording area. (**B**) Pseudocolor images showing fluorescence intensity on the ventral medulla before, during, and after tetanic stimulation. (**C**) Time course of fluorescence intensity in the red square area of panel B-a. A horizontal red bar shows the stimulation period. **a**: onset of stimulation, **b**: end of stimulation, **c**: 5.0 s after the end of stimulation, **d**: 10.0 s after the end of stimulation, **e**: 15.0 s after the end of stimulation, **f**: 20.0 s the end of after stimulation. The excitations of the RVLM, CVLM, RVMM and RP were well detected. RVLM, rostral ventrolateral medulla; CVLM, caudal ventrolateral medulla; RP, raphe pallidus.

In response to tetanic stimulation of the DMH, neural activation was detected in the RVLM, RVMM, and RP in all specimens, whereas activation of the CVLM was seen in four out of the five specimens (Fig. 5).

**Figure 6:**
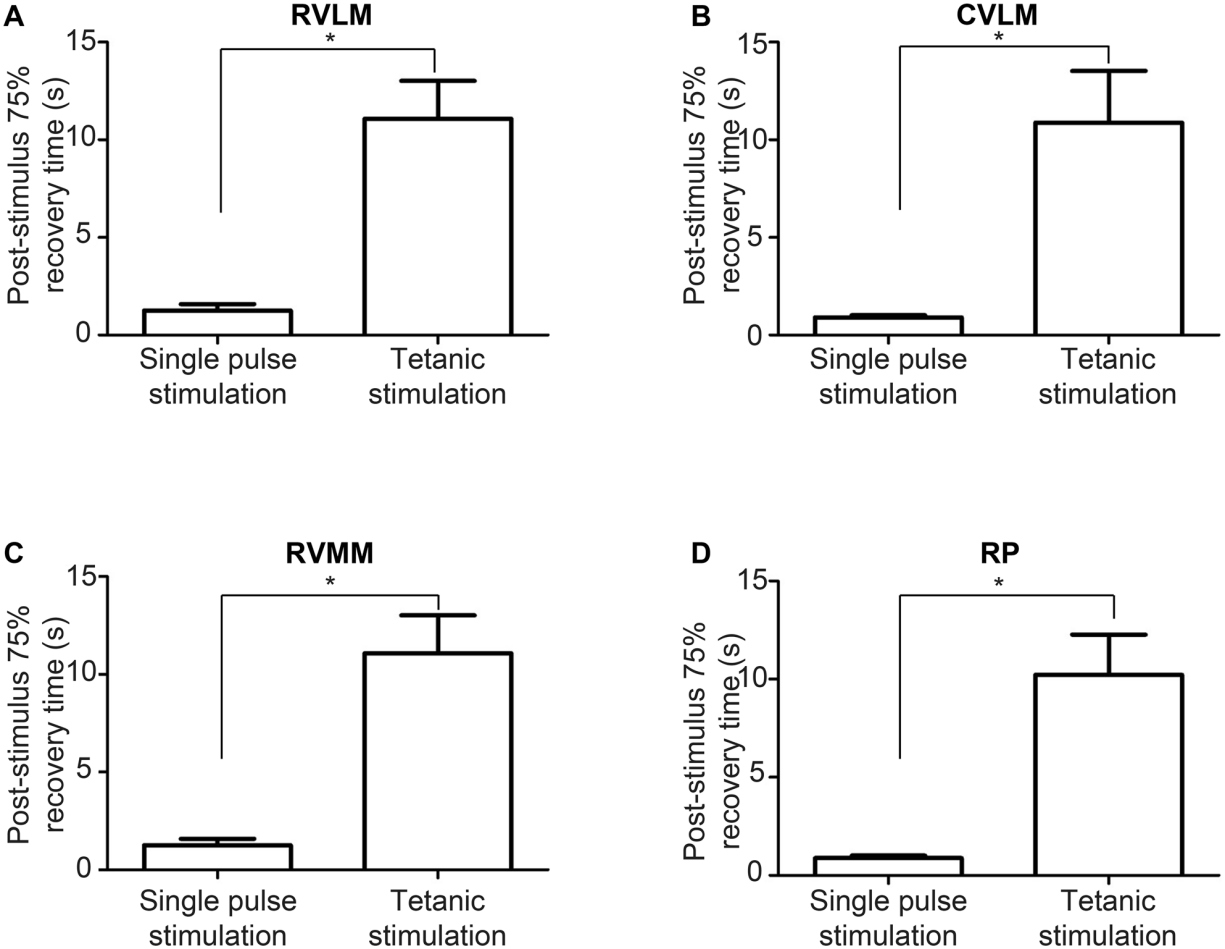
Comparison of post-stimulus 75% recovery time in single pulse stimulation and in tetanic stimulation in (**A**) RVLM, (**B**) CVLM, (**C**) RVMM, and (**D**) RP. RVLM; rostral ventrolateral medulla, CVLM, caudal ventrolateral medulla; RVMM, rostral ventromedial medulla; RP, raphe pallidus. *p < 0.05.

Of note, tetanic, but not single pulse, stimulation of the DMH induced a delayed recovery from the RVLM activation after cessation of stimulation. In tetanic stimulation, post-stimulus 75% recovery time was significantly longer, as compared with that in single pulse stimulation, in the RVLM, CVLM, RVMM, and RP areas (RVLM: 1.26 ± 0.32 vs. 11.08 ± 1.95 s, CVLM: 0.91 ± 0.12 vs. 10.88 ± 2.65 s, RVMM: 1.19 ± 0.29 vs. 10.30 ± 2.22 s, RP: 0.89 ± 0.12 vs. 10.22 ± 2.06 s, respectively. *p* < 0.05 for all) (Fig. 6).

## Discussion

In this study, tract-tracing revealed that DMH neurons send axonal projection directly to the ventral medullary cardiovascular regions and that some DMH neurons terminate onto dendrites of TH-immunoreactive neurons in the RVLM. In voltage imaging, both single pulse and tetanic electrical stimulation of the DMH induced depolarizing neural activity in the ventral medullary cardiovascular regions. However, recovery of medullary activity after cessation of stimulation was remarkably delayed after tetanic, but not after single pulse stimulation, indicating that the connectivity from the dorsomedial hypothalamus to the medullary cardiovascular networks serves as a chronological amplifier of stress-induced sympathetic excitation. The ventral medullary regions to which DMH neurons sent axonal projections coincided with the regions depolarized by stimulation of the DMH in voltage imaging experiments.

The major finding of this study is that the DMH and several ventral medullary regions that are important in cardiovascular regulation (RVLM, CVLM, RVMM and RP) are anatomically and functionally connected. Further, we anatomically confirmed for the first time that DMH neurons directly innervate catecholaminergic neurons in the RVLM. Several other histological studies have also reported a monosynaptic connection from the DMH to the dorsal motor nucleus of the vagus, nucleus ambiguus, and parvocellular reticular formation of the medulla^20^. Lindberg et al.^21^ have shown synaptic contacts between the ventromedial hypothalamic neuronal efferents and TH-positive catecholaminergic neurons in the RVLM and the nucleus of the solitary tracts. However, the connectivity between the DMH efferents and TH-immunopositive neurons in the RVLM or other cardiovascular regions in the medulla have not been reported. In acute stress, the DMH plays a vital role in integrating autonomic, neuroendocrine, and behavioral responses^5^. Lisa et al.^22^ have demonstrated that inhibition of the DMH abolishes the stress-induced cardiovascular response. Sympathetic premotor neurons in the RVLM are excited by DMH disinhibition^7,23^. Also, medullary raphe neurons mediate sympatho-excitation evoked by DMH disinhibition^7,24^. While we did not measure blood pressure or heart rate, we, as well as the above mentioned previous studies, have demonstrated that hypothalamic stimulation excitates the ventral medullary areas that are involved in stress-induced cardiovascular responses. These findings help understand how the ventral medulla is activated by acute psychological stress.

In this study, tetanic hypothalamic stimulation excited not only the RVLM, RVMM and RP but also the CVLM. Our tract-tracing experiments revealed the anatomical connectivity from the DMH to the CVLM. It is well established that arterial baroreceptor afferents are excited by blood pressure elevation. They project to the nucleus of the solitary tract and provide a major excitatory input to GABAergic CVLM neurons. These neurons, in turn, inhibit the activity of presympathetic RVLM neurons^25^. Thus, the CVLM has a sympatho-inhibitory role. However, it has also been reported that neurons in the caudal pressor area (CPA), which is located just caudal to the CVLM, are sympatho-excitatory^26,27^. CPA neurons mediate the pressor response by inhibiting RVLM-projecting inhibitory units and activating RVLM-projecting excitatory CVLM units^27^. Among the ventral medullary regions activated by DMH stimulation, which was detected by our voltage imaging, the most caudal area could correspond to the CPA, suggesting the possible excitatory innervation of the CPA by the DMH.

It is noteworthy that tetanic stimulation of the hypothalamus induced post-stimulus sustained excitation in the ventral medulla, plausibly related to blood pressure regulation. However, our observations do not address the exact mechanism of delayed post-stimulus recovery. In the medullary cardiovascular regions, there exist a high density of angiotensin II type 1 receptors^28,29^. Glass et al.^30^ have reported that angiotensin II-induced blood pressure elevation could be a phenotype of neural plasticity. Recent studies have demonstrated that not only neurons but also glial cells, especially astrocytes, are critically involved in neural plasticity^19,31,32^. Neurotransmitters, released from presynaptic terminals, activate nearby astrocytes in a paracrine manner. The activated astrocytes augment neuronal synaptic transmission by releasing gliotransmitters^33^. These phenomena occur within a few seconds but could persist for more than several minutes. Thus, astrocytes may also be involved in the blood pressure elevation evoked by psychological stress and, particularly, in the post-stress sustained blood pressure elevation. Voltage imaging could detect both neuronal and astrocytic excitation^19,34^. However, in the present study we could not distinguish optical signals from neurons and astrocytes. Further studies are needed to clarify the distinctive roles of neurons and astrocytes in the hypothalamic activation-induced sustenance of ventral medullary excitation, using different experimental designs, for instance, by calcium imaging.

Acute psychological stress induces elevation of blood pressure, which persists after the stress is relieved^35^. Recently, several studies have reported that the time course of post-stress cardiovascular recovery is predictive of future hypertension and other cardiovascular events^1,36,37^. Thus, a study of post-stress cardiovascular recovery contributes to the elucidation of the pathophysiology of hypertension. Our finding that tetanic stimulation of the hypothalamus induces post-stimulus sustained activation of the ventral medullary cardiovascular regions indicates that the connectivity from the DMH to the medullary cardiovascular networks serves as a chronological amplifier of stress-induced sympathetic excitation, and gives insight into the mechanisms underlying the persistence of post-stress sympathoexcitation.

Among the medullary regions that are anatomically and functionally connected with the DMH, the RVMM is also important in processing nociceptive information including both antinociception and pronociception^38,39^. Wagner et al. reported that the DMH mediates stress-induced hyperalgesia via the RVMM^39^. Stress-induced DMH excitation may contribute to pain-induced blood pressure elevation via the activation of medullary regions including the RVMM, and this issue must be further clarified.

There may be a criticism that the tetanic stimulation-induced depolarizing activity in the ventral medulla is simply non-specific post-tetanic facilitation. However, in our number of preliminary trials of stimulating the diencephalon at the DMH as well as at many other nearby sites other than the DMH, we did not obtain positive responses to stimulation of sites other than the DMH. Thus, the tetanic stimulation-induced depolarizing activity in the ventral medulla could not be non-specific post-tetanic facilitation.

The experimental method of the present study has some limitations. Voltage imaging in the isolated diencephalon-lower brainstem-spinal cord preparation is performed in the condition devoid of blood circulation, in which the tissue PO_2_ and pH environment are maintained within the physiological range in the layer only up to several hundred micrometers from the surface^40^. However, the function of the sympathetic neural network of the ventral medulla and spinal cord is reported intact in the preparation^41^. Further, it has been confirmed that the respiration-facilitating function of the diencephalon also remains intact in this preparation^13,42^. To further improve hypothalamic tissue oxygenation, we removed the brain structures rostral to the point of stimulation (Fig. 1). In this study, we performed electrical stimulation of the DMH in the neonatal rat preparation to mimic psychological stress. It has been reported that functional connectivity from the ventral medullary cardiovascular regions to the intermediate lateral cell column of the spinal cord is also intact in the neonatal rat brainstem-spinal cord preparation^41,43^. Therefore, it is conceivable that the use of our in vitro preparation is suitable for the evaluation of functional connectivity between the hypothalamic and ventral medullary cardiovascular regions.

Although we obtained interesting findings by recording depolarizing activities in the cardiovascular regions in the ventral medulla, neither sympathetic nerve activity nor blood pressure was recorded in this reduced preparation, which is a major limitation when interpreting these results. The functional experimental findings obtained in the present study should be confirmed in in vivo experiments by monitoring sympathetic nerve output and blood pressure.

Delayed recovery of ventral medullary excitation after cessation of tetanic stimulation of the hypothalamus, as observed in the present study, may have to do with the post-stress persistent sympathetic activation and the development of hypertension. In the present study, we stimulated the hypothalamus only for 10 s. Chronic psychological stress usually acts over a longer time scale of months or even years, resulting in hypertension. Nonetheless, Hall^44^ has indicated that stress affects blood pressure also in a matter of seconds and the accumulation of short sympathetic responses underlies the development of hypertension. The mechanism of post-stimulus sustained excitation in the ventral medulla should be investigated over different time scales to better understand the pathophysiology of stress-induced hypertension.

Elucidation of the post-stress autonomic neural responses could provide a clue for the pathophysiologic mechanism of stress-induced hypertension. In this study, we demonstrated that neurons in the DMH send axonal projections to the ventral medullary cardiovascular regions. Besides, electrical stimulation of the DMH induces neural excitation in the ventral medullary cardiovascular regions and recovery from the excitation is delayed after tetanic stimulation is applied. We identified ventral medullary areas that are anatomically and functionally connected with the DMH. Thus, the connectivity from the DMH to the medullary cardiovascular networks serves as a chronological amplifier of stress-induced sympathetic excitation. To further understand the hypothalamus-ventral medullary connectomics, cells and transmitters involved should be investigated, with attention directed toward astrocytes and angiotensin II.

## Supplementary information

Supplementary file1

## Data Availability

The data that support the findings of this study are available from the corresponding author upon reasonable request.

## Code availability

The voltage imaging data analysis software, BV Analyzer, can be obtained from BrainVision (Tokyo, Japan).
