# Supplementary material for: Structural and functional connectivity from the dorsomedial hypothalamus to the ventral medulla as a chronological amplifier of sympathetic outflow
Source: Sci Rep. 2020 Aug 7;10:13325. doi: 10.1038/s41598-020-70234-4 (PMC7414200; doi:10.1038/s41598-020-70234-4)
Supplement: Supplementary file 1 — Supplementary file1 [file 41598_2020_70234_MOESM1_ESM.pdf]

Supplementary Information

Structural and functional connectivity from the dorsomedial hypothalamus to the ventral medulla as a chronological amplifier of sympathetic outflow

Yosuke Kono[1,2], Shigefumi Yokota[3], Isato Fukushi[2,4], Yosuke Arima[3], Hiroshi Onimaru[5], Shuntaro Okazaki[6], Kotaro Takeda[7], Itaru Yazawa[8], Masashi Yoshizawa[1,2], Yohei Hasebe[1,2], Keiichi Koizumi[1], Mieczyslaw Pokorski[9], Takako Toda[1], Kanji Sugita[1], Yasumasa Okada[2*]

[1] Department of Pediatrics, Faculty of Medicine, University of Yamanashi, Yamanashi, 409-3898, Japan

[2] Clinical Research Center, Murayama Medical Center, Tokyo, 208-0011, Japan

[3] Department of Anatomy and Morphological Neuroscience, Shimane University School of Medicine, Izumo, 693-8501, Japan

[4] Faculty of Health Sciences, Uekusa Gakuen University, Chiba, 264-0007, Japan

[5] Department of Physiology, Showa University School of Medicine, Tokyo, 142-8555, Japan

[6] Faculty of Human Sciences, Waseda University, Tokorozawa, 359-1192, Japan

[7] Faculty of Rehabilitation, School of Healthcare, Fujita Health University, Toyoake, 470-1192, Japan

[8] Global Research Center for Innovative Life Science, Hoshi University, Tokyo, 142-8501, Japan

[9] Faculty of Physiotherapy, Opole Medical School, Opole, 45-060, Poland

Equal contribution: Yosuke Kono and Shigefumi Yokota contributed equally to the present study.

* For correspondence: Yasumasa Okada, MD, PhD

Clinical Research Center, Murayama Medical Center, 2-37-1 Gakuen, Musashimurayama, Tokyo 208-0011, Japan

Tel: +81-42-561-1221, Fax: +81-42-564-2210

Email: yasumasaokada@1979.jukuin.keio.ac.jp

Supplementary Figure 1

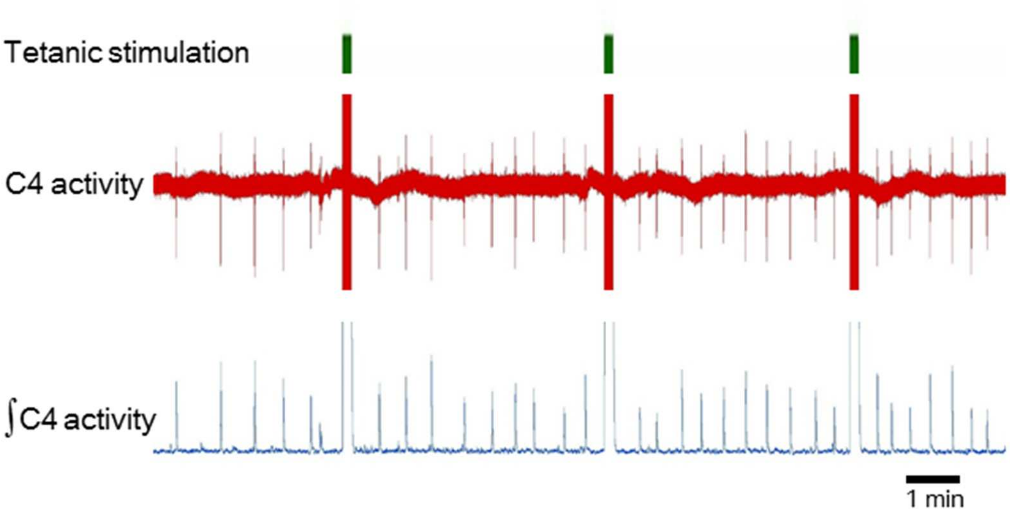

Raw C4 activity and its integrated signal traces during a tetanic stimulation experiment for the DMH. Viability of the preparation was confirmed by the stable neural respiratory activity recorded from C4 ventral root of the cervical spinal cord.

Supplementary Figure 2

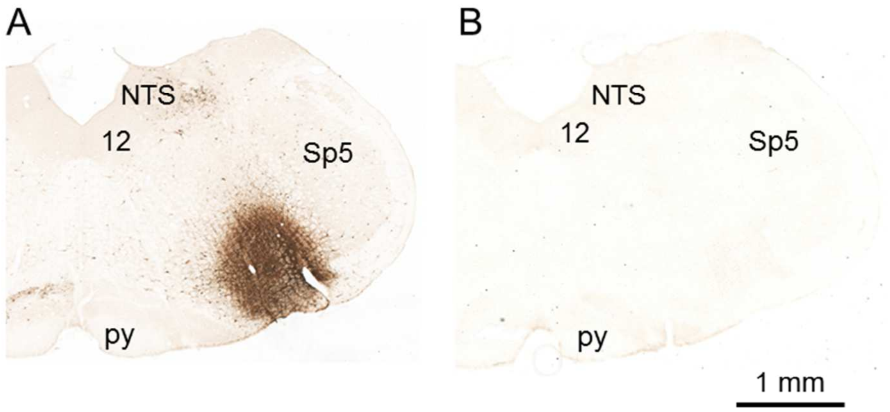

Negative control for guinea pig anti-FG antibody. We used two series of sections from another rat received FG injection into the rostral ventrolateral medulla (RVLM). The sections were immunostained with or without guinea pig anti-FG antibody. **(A)** Immunostained with primary antibody. **(B)** Immunostained without primary antibody. The immunostaining with primary antibody shows and injection site in the RVLM as well as retrograde cell labeling in the NTS, whereas the immunostaining without the primary antibody shows no labeling. NTS, nucleus of the solitary tract; py, pyramidal tract; Sp5, spinal trigeminal nucleus; 12, hypoglossal nucleus.

Supplementary Figure 3

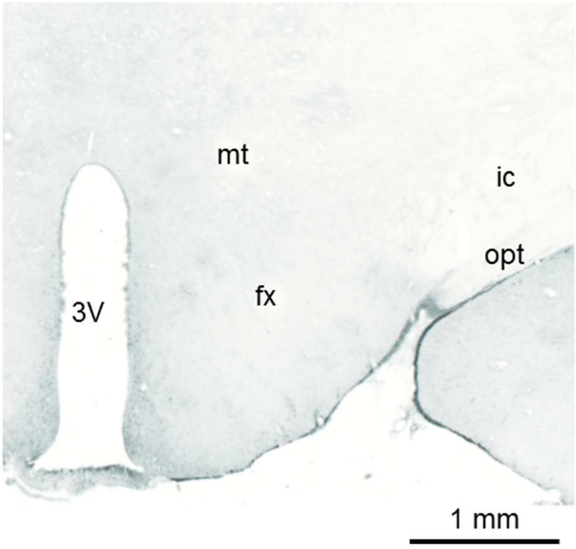

Negative control for BDA histochemistry. We used brain sections from the rat received no injection of BDA into any brain area. We conducted the same procedures in the sections as an anterograde tracing experiment. We found no labeling either in the hypothalamus or in any other brain area. fx, fornix; ic, internal capsule; mt, mammillothalamic tract; opt, optic tract; 3V, third ventricle.

Supplementary Figure 4

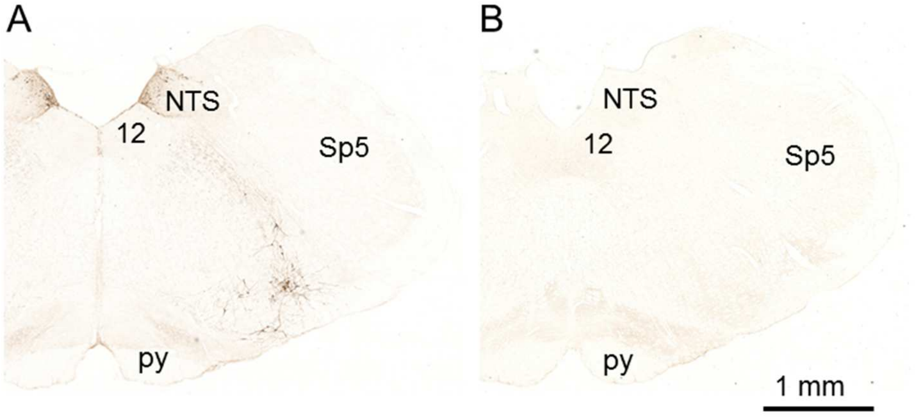

Negative control for mouse anti-TH antibody. Two series of the sections were immunostained with or without mouse anti-TH antibody. **(A)** Immunostained with primary antibody. **(B)** Immunostained without primary antibody. The immunostaining with primary antibody shows immunoreactive cells in the RVLM and NTS which are defined as A1/C1 and A2/C2 catecholaminergic cell groups, respectively. On the other hand, no immunoreactivity is found without the primary antibody. For abbreviations, see the legend of the supplementary Figure 2.

Supplementary Figure 5

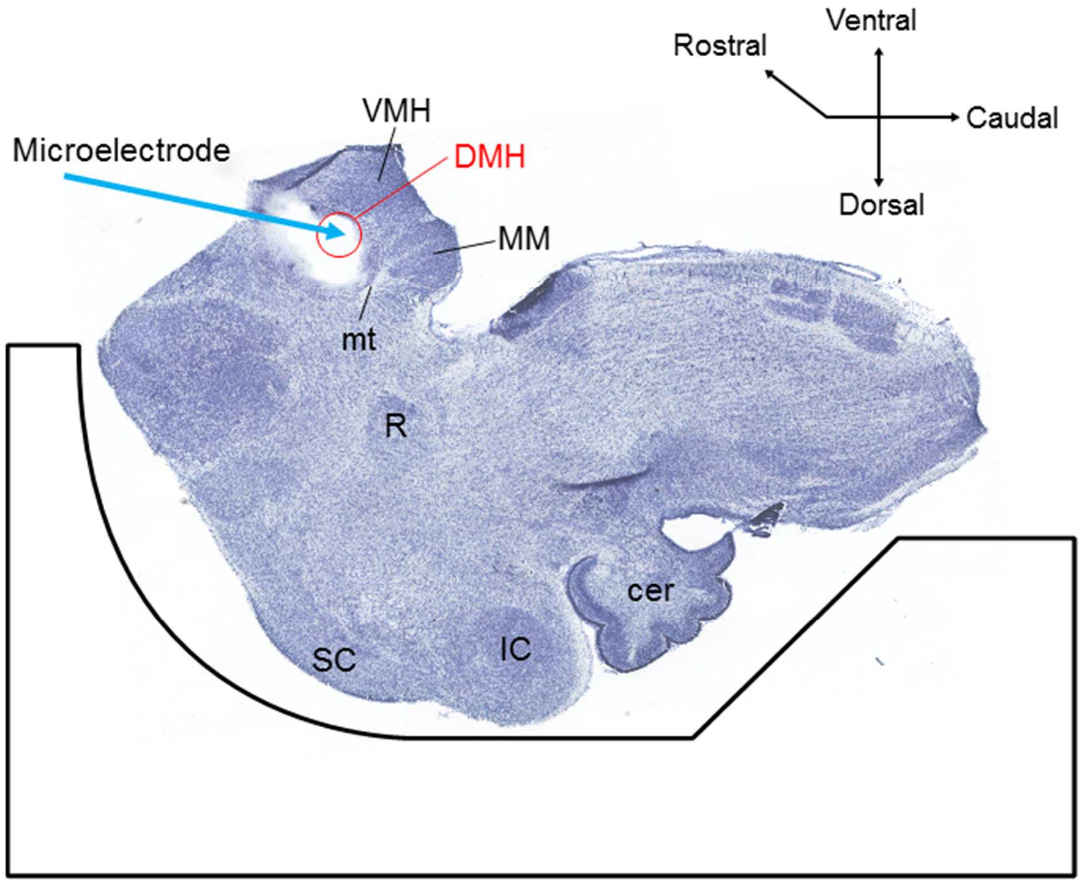

The site of stimulating microelectrode placement in the hypothalamus. At the end of imaging experiments, the location of the electrode tip was marked by passing 5 mA direct current for 3 s. After fixed with formalin, 50 μm thick sagittal sections were cut and then stained with 1% cresyl violet for cytoarchitectural landmarks. A cavitary lesion was formed with electrolytically generated gas bubble that expanded the region dorsal to the VMH. Note that, as described above, the cavity is formed with a gas bubble, and the cauterized brain region is much smaller than the cavity and corresponds to the DMH. Thus, the accuracy of the stimulating microelectrode placement was histologically confirmed. MM, medial mamillary nucleus; mt, mamillothalamic tract; VMH, ventromedial nucleus of the hypothalamus; R, red nucleus. For anatomical orientation, see Figure 1A. Note that the angle between the longitudinal axes of the diencephalon and of the lower brainstem in this panel is different from that in Figure 1.
